# Successful pregnancy after prenatal diagnosis by NGS for a carrier of complex chromosome rearrangements

**DOI:** 10.1186/s12958-020-00572-5

**Published:** 2020-02-29

**Authors:** Jian Ou, Chuanchun Yang, Xiaoli Cui, Chuan Chen, Suyan Ye, Cai Zhang, Kai Wang, Jianguo Chen, Qin Zhang, Chunfeng Qian, Guangguang Fang, Wenyong Zhang

**Affiliations:** 1 Center for Reproduction and Genetics, The Affiliated Suzhou Hospital of Nanjing Medical University, Suzhou Municipal Hospital, Suzhou, 215002 China; 2 CheerLand Biological Technology Co., Ltd, Shenzhen, 518000 China; 3 Shenzhen Dapeng New District Maternity & Child Health Hospital Department of Gynecology, Shenzhen, China; 4 Shenzhen Second People’s Hospital, The First Affiliated Hospital of Shenzhen University, Shenzhen, China; 5 Southern University of Science and Technology-CheerLand Institute of Precision Medicine, Shenzhen, China; 6 School of Medicine, Southern University of Science and Technology, Shenzhen, China

**Keywords:** Complex chromosomal rearrangements, Breakpoints mapping, Whole-genome low-coverage mate-pair sequencing, Preimplantation genetic testing for aneuploidy, Junction-spanning PCR

## Abstract

**Background:**

The study is aimed to provide prediction for fertility risk in the setting of assisted reproduction for a woman with complex chromosomal rearrangements (CCRs).

**Methods:**

We implemented a robust approach, which combined whole-genome low-coverage mate-pair sequencing (WGL-MPS), junction-spanning PCR and preimplantation genetic testing for aneuploidy (PGT-A) method to provide accurate chromosome breakpoint junctional sequences in the embryo selection process in the setting of assisted reproduction for a couple with recurrent abortions due to CCRs.

**Result:**

WGL-MPS was applied to a female carrying CCRs which consisted of 9 breakpoints and 1 cryptic deletion related to fertility risks. Sequencing data provided crucial information for designing junction-spanning PCR and PGT-A process, which was performed on the 11 embryos cultivated. One embryo was considered qualified for transplanting, which carried the exact same CCRs as the female carrier, whose phenotype was normal. The amniotic fluid was also investigated by WGL-MPS and karyotyping at 19 weeks’ gestation, which verified the results that the baby carried the same CCRs. A healthy baby was born at 39 weeks’ gestation by vaginal delivery.

**Conclusion(s):**

Our study illustrates the WGL-MPS approach combining with junction-spanning PCR and PGT-A is a powerful and practical method in the setting of assisted reproduction for couples with recurrent miscarriage due to chromosomal abnormalities, especially CCRs carriers.

## Background

Complex chromosomal rearrangements (CCRs) are structural rearrangements involving three or more cytogenetic breakpoints on more than two chromosomes [1, 2]. It has been estimated that 3.5% of couples with a history of recurrent miscarriage have at least one partner who is a carrier of a chromosomal structural rearrangement [3]. The most frequent of these rearrangements is translocation. Other rearrangements include inversions, insertions, deletions, duplications, or, rarely, ring chromosomes [4]. The potential risk for chromosome imbalance in the gametes of CCRs carriers is higher than those with simple translocations, and thus contributing to higher risk of recurrent miscarriage [5]. The incidence of spontaneous abortions and abnormal pregnancy outcomes in CCRs families was estimated to be 48.3 and 53.7%, respectively [6]. Almost 18.4% of all live births from CCRs carriers result in phenotypically abnormal offspring and one-half of all CCRs carriers produce offspring who are also CCRs carriers [6]. Moreover, the higher the complexity of CCRs the higher the risk for unbalanced gamete generation and hence the higher the risk for having an affected offspring [7, 8]. In order to assess the risk faced by CCRs carriers who consider pregnancy as accurately as possible, precise characterization of CCRs is of crucial importance.

Several cytogenetic and molecular methods such as Giemsa banding, fluorescence in situ hybridization (FISH), array-comparative genomic hybridization and array painting have been applied to study chromosomal structural changes associated with abnormal phenotypes [9]. However, these techniques lack the precision that is required to define the rearrangement at nucleotide level, may fail in identifying smaller chromosomal duplications and deletions, and are often technically challenging and time consuming to perform [10–12].

In recent years, a robust method for global detection of balanced chromosomal rearrangements by whole-genome low-coverage mate-pair sequencing (WGL-MPS) has been developed for detailed investigation of CCRs [13]. The approach can identify nearly all cryptic chromosomal abnormalities or complex rearrangements present in the genome. In addition, it is capable of characterizing translocation breakpoints at the nucleotide level [12–15]. Therefore, this method is of value to provide prenatal genetic counseling for couples with reproductive issues by comprehensively mapping CCRs and providing precise breakpoint sequences for subsequent PGT-A.

## Methods

### Case presentation

**Fig. 1:**
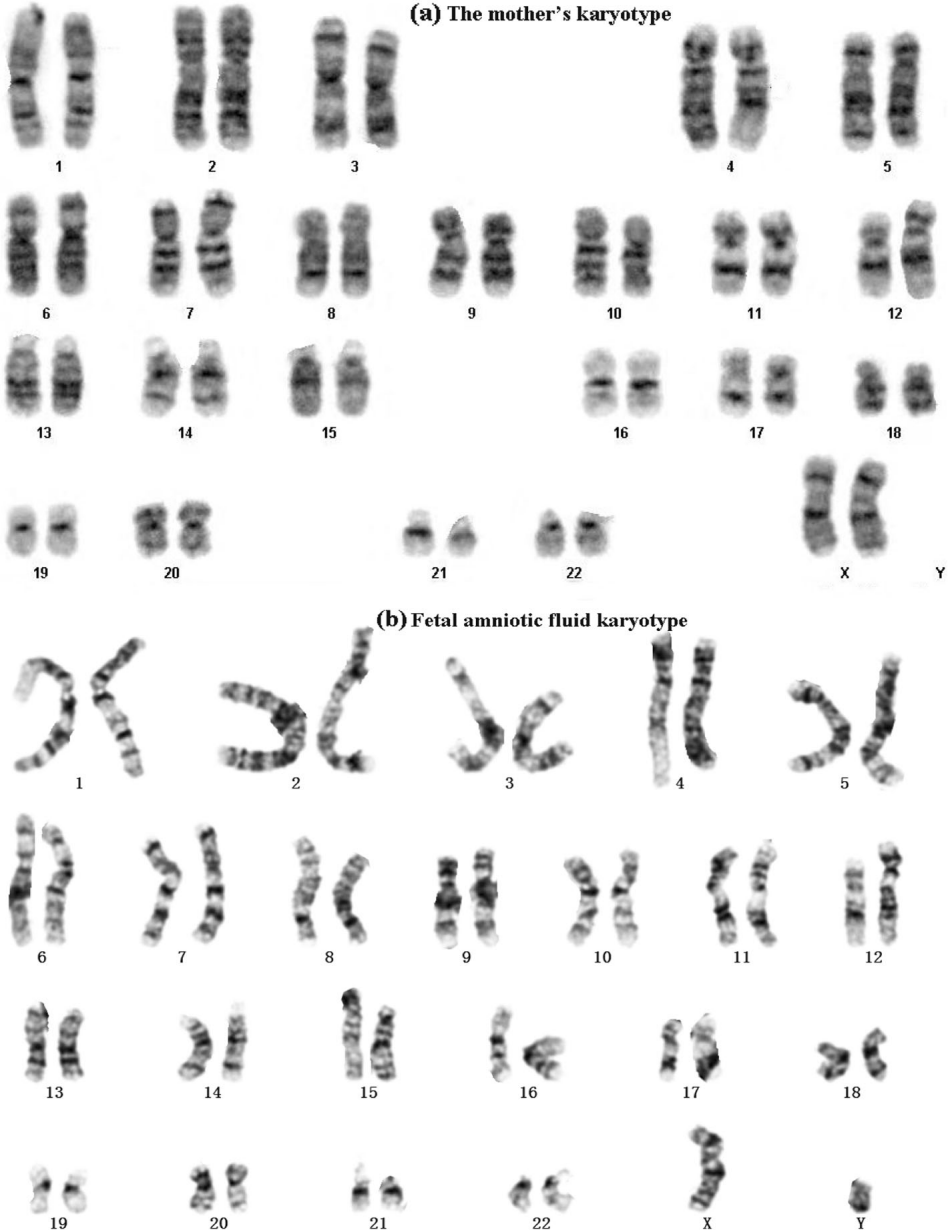
Maternal and fetal karyotype. (**a**) Mother karyotype. (**b**)19-week fetal amniotic fluid karyotype. Their karyotypes are 46, XX, der(1)t(1:4)(p22:q31.1),der(4)ins(5:4)(q22;q25q28)t(1:4), der(5)ins(5:4)

A young couple (woman and man 27 and 30 years old, respectively) experienced two consecutive early spontaneous miscarriages. The cause of infertility was unknown. Karyotyping was performed on G-banded metaphase spreads of cultured lymphocytes using conventional methods. The man had normal 46, XY karyotype, while the woman was found to carry complex chromosome rearrangement: a q25q28 fragment of chromosome 4 was inserted into q22 in chromosome 1, and this chromosome 4 was shifted in equilibrium with chromosome 5. The breaking points were on 4q31.1 and 1q22, respectively. Her karyotype (Fig. 1) is:

46, XX, der(1)t(1:4)(p22:q31.1),der(4)ins(5:4)(q22;q25q28)t(1:4),der(5)ins(5:4).

### WGL-MPS analysis and breakpoint validation

According to the results of karyotyping analysis, there was very little possibility for her to give birth to a normal child through natural pregnancy and she faced with an increased risk for having an affected offspring.

To make sure the exact location of the breakpoint and learn more about the risks of abnormal pregnancy outcomes, WGL-MPS was performed on the woman. Her genomic DNA was extracted from peripheral blood with Qiagen DNA extraction kit and then used to construct a non-size selected mate-pair library [12] and then subjected to 50-bp-end multiplex sequencing by BGISeq-500. After removing reads containing sequencing adapters and low-quality reads, the high-quality pair-end reads were aligned to the NCBI human reference genome (hg19, GRCh37.1) using SOAP2. Only uniquely mapped reads were remained for the subsequent analysis as previously described [13, 15]. The breakpoints were validated by junction-spanning PCR as previously described [9]. The PCR primer pairs were reserved sufficiently.

### Preimplantation genetic testing for aneuploidy

The woman used a long protocol, or a GnRH (Gonadotropin-releasing hormone) antagonist protocol for controlled ovarian hyperstimulation. Oocytes were retrieved 34 to 35 h after hCG injection and fertilized with intracytoplasmic sperm injection (ICSI). we obtained 20 eggs through two cycles and 15 eggs were successfully fertilized, and 11 eventually developed into blastocysts. The ovarian stimulation, oocyte retrieval and embryos culture were performed as described by Yanagimachi R, et al [16]. The trophectoderm cells from the blastocysts were obtained as described by Jian Ou, et al [17], and rinsed three time with G-MOPS (Vitrolife) medium, and then transferred to RNAse–DNAse-free PCR tubes (Axygen) with the minimum medium*.* Whole genome amplification (WGA) was performed using a QIAGEN kit. Amplification products were stored at − 20 °C. To avoid contamination, this process should be all handled in a ventilation cabinet. The breakpoints validation was performed on the amplification products with the PCR primer pairs kept previously and only three embryos (including two embryos with 9 breakpoints inherited and one embryo without breakpoints) were kept for further analysis. PGT-A was done by comprehensive chromosomal screening on these three embryos [17]. An embryo was found to be a balanced euploid and transferable. After genetic counseling, the couple decided to go ahead with implantation. The HCG level was tested 14 days after the embryo transfer. Pregnancy was confirmed by fetal heartbeat on ultrasonography. Amniocentesis at 19 weeks’ gestation was performed to confirm prenatal diagnosis.

## Results

**Fig. 2:**
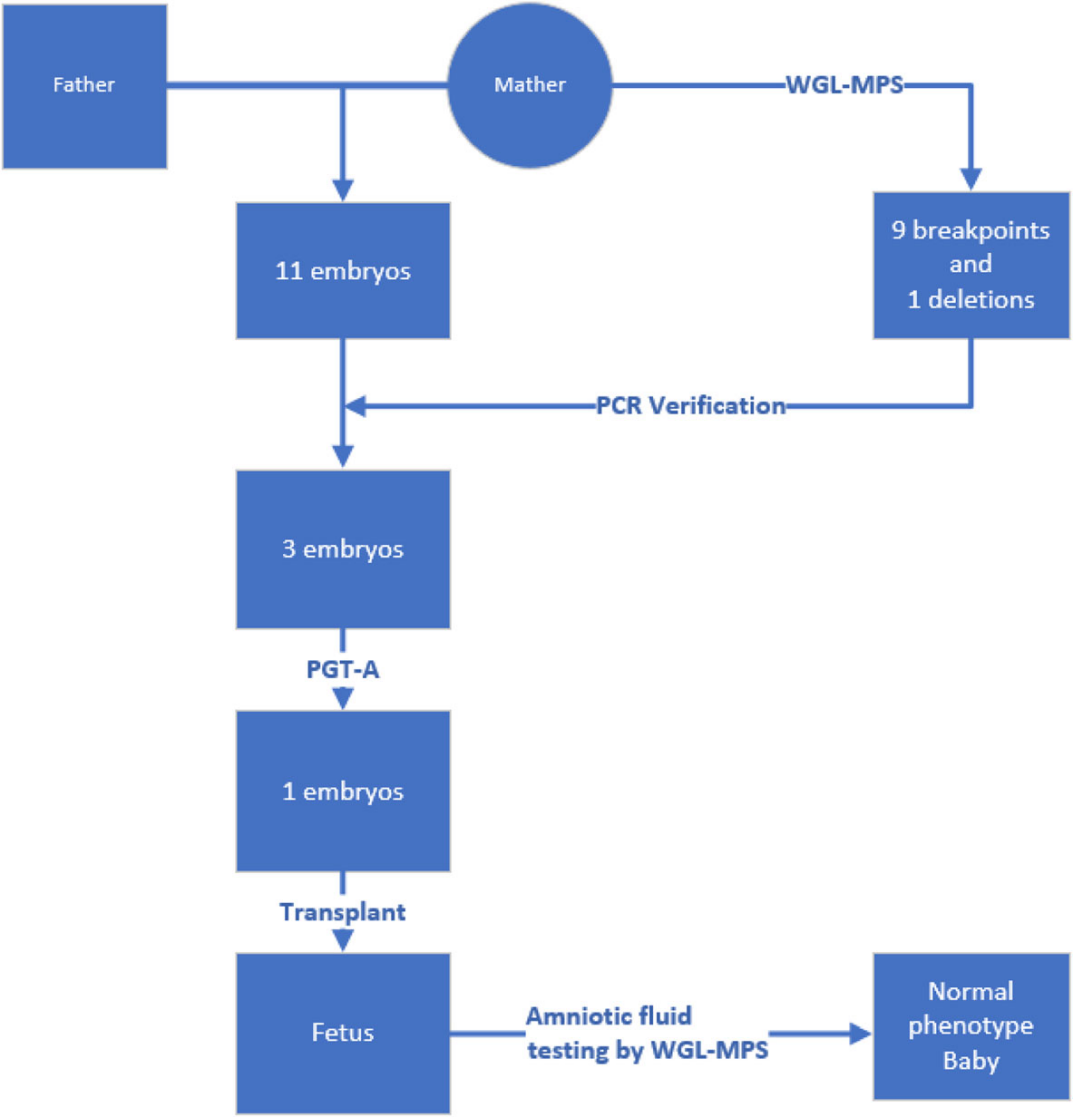
Experimental operation flow chart. First, we used WGL-MPS technology to detect the CCRs in maternal chromosomes. Secondly, we used PCR to verify the corresponding breakpoints of the 11 embryos generated by serial oocyte verification. Third, we performed PGT-A testing on the selected 3 embryos, and finally obtained an embryo with the same CCRs as the mother. Finally, we transplanted a screened qualified embryo and a normal phenotype baby with the same CCRs as its mother was born

In this study, we presented a unique case of a woman diagnosed with very complex chromosomal rearrangements whose corresponding breakpoints were precisely identified by WGL-MPS. We used junction-spanning PCR to verify the corresponding breakpoints of the embryos generated during assisted reproduction and further checked for aneuploidy by conventional PGT-A. After careful counseling and obtaining consent from the couple, we transplanted a screened qualified embryo and a normal phenotype baby with the same CCRs as its mother was born. Here we describe such approach (Fig. 2)in the clinical setting.

**Fig. 3:**
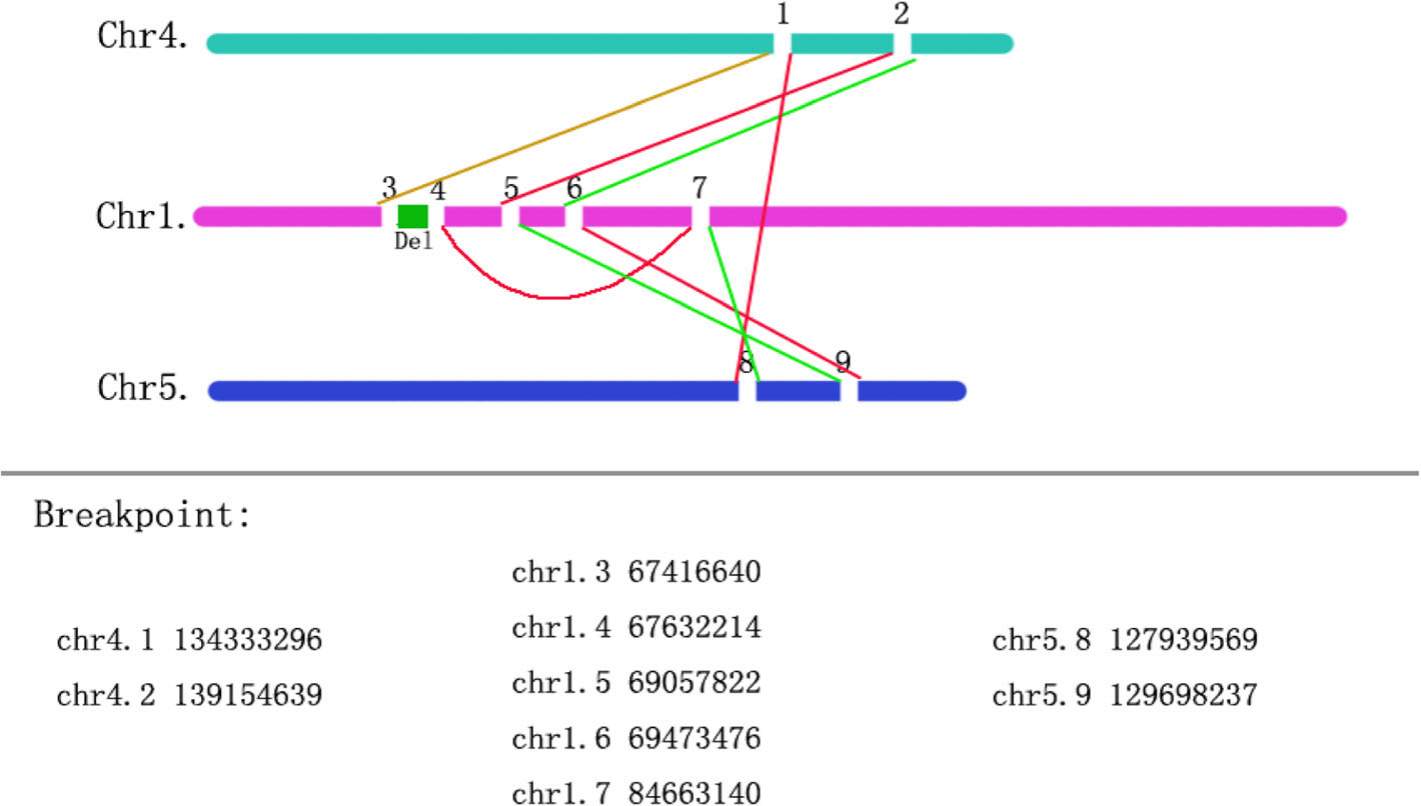
Reassembly of all chromosomal regions that were involved in the translocations, according to HG19 (www.genome.ucsc.edu)

G-banding analysis at a band resolution of ∼400 revealed the woman to be a carrier of balanced translocation among the three chromosomes and the two breakpoints were on 4q31.1 and 1p22, respectively. However, WGL-MPS analysis indicated a far more complicated rearrangement. In summary, 9 breakpoints and a microdeletion on chromosome 1 were identified as showed in Fig. 3. Using the new nomenclature for sequenced breakpoints proposed by Ordulu [18], The formula for the chromosome translocation was thus revised as:

46,XX, der(1)ins(1;4)(1qter- > 1p31.1 (5q23.3::1p31.2) 4q28.3- > 4qter),der(4)t(4:1).

(4pter- > 4q31.1::1p28.3- > 1pter), der(5)ins(5)(5pter- > 5q23.3(t(4,1)(4q28.3(inv(1)

(p31.3::p31.2) inv.(1)(p31.2::p31.1)) 5q23.3- > 5qter).

In our study, four genes, including *C1orf141, IL23R, MIER1, SLC35D1* are disrupted at the deletion on 1p31.3. The *IL23R* gene provides instructions for making a protein called interleukin 23 (IL-23) receptor. Sequence variations in *IL23R* gene have also been associated with the risk of several other immune system-related conditions, like psoriasis and inflammatory bowel disease. *SLC35D1* is a nucleotide sugar transporter that localizes to the endoplasmic reticulum and transports both UDP-glucuronic acid and UDP-N-acetyl galactosamine. Homozygous and compound heterozygous loss-of-function *SLC35D1* mutations have been reported in patients with Schneckenbecken dysplasia. On chromosome 1, the *PRKACB* gene encoding a catalytic subunit of cAMP-dependent protein kinase (PKA) is interrupted at the 7th breakpoint. On chromosome 4, the *SLC7A11* gene is disrupted at the 2nd breakpoint. On chromosome 5, *FBN2* and *SLC27A6* are disrupted at the 8th breakpoint. The *FBN2* gene, encoding a large protein called fibrillin-2, is annotated in OMIM to be associated with autosomal dominant congenital contractual arachnodactyly and early-onset macular degeneration. Fortunately, the woman is not affected by the 8th breakpoint, probably because the breakpoint is close to the end of *FBN2* gene sequence. No other known gene is interrupted by the remaining breakpoints, which are breakpoint 1, breakpoint 5, breakpoint 6 and breakpoint 9.

Eight pairs of primers were designed according to flanking sequences of the breakpoints. The sequences of the primers were displayed in Table 1. If the breakpoints location and sequences were predicted correctly as showed in Fig. 3 and the primers were valid, the corresponding bands of the amplification products should be presented on the electropherogram.

**Table 1:** Primer information of the breakpoints

Primer Name	Primer pair	Sequence (5′- > 3′)	Length	Tm	GC%	Product length [19]
P1	Forward primer	GGCTGGGAAGTCCAACACGA	20	62.39	60	382
	Reverse primer	CTAGTTCAGTCTGGATGTGGTCC	23	60.12	52.17	
P2	Forward primer	GGCAACCTAATCAAGTACGGAA	22	58.4	45.45	471
	Reverse primer	CTCTCTTGCCTCACAAATGCAC	22	60.1	50	
P3	Forward primer	GGGAAGAGCCTTGCTCGTA	19	59.1	57.89	336
	Reverse primer	TAAAGCAGGTATGCGTGAGATTG	23	59.13	43.48	
P4	Forward primer	GACAAAATGACAGCAATAAGCCC	23	58.82	43.48	320
	Reverse primer	AGTTGGAAATCCTTCCTCAACTC	23	58.34	43.48	
P5	Forward primer	TGCAGGTTAAGTCCTCCGTTT	21	59.58	47.62	421
	Reverse primer	GGGTTATGTTACCCTTCTGCCTAA	24	60.08	45.83	
P6	Forward primer	TGTAGTGGCACGATCTCAGC	20	59.83	55	351
	Reverse primer	TCCTTGCCTCTTCCATTTGT	20	57.02	45	
P7	Forward primer	GCATGGCTCATCATATCGCATAA	23	59.38	43.48	473
	Reverse primer	TGATGGTGCAACTAATGGCAGA	22	60.29	45.45	
P8	Forward primer	CTGGCTCAACTTTTGATGAGTGT	23	59.43	43.48	391
	Reverse primer	TTCCAGAGAGTGGGGTCATCT	21	59.92	52.38	

The WGA product of trophectoderm cells from eleven embryos underwent breakpoint analysis using PCR primer pairs designed to amplify junctional sequences and three embryos (including two embryos with 9 breakpoints inherited and one embryo without breakpoints) underwent the PGT-A protocol. PGT-A showed that Embryo4 was chr16 triploid and Embryo9 had a 6q16.1(93,100,000-99,500,000) deletion (Table 2). A single euploid embryo, identified to carry all the same nine breakpoints as its mother was implanted. Prenatal diagnosis by amniocentesis and WGL-MPS was performed at 19 weeks’ gestation, which revealed the fetus to be a carrier of the same complex chromosomal rearrangements and deletion as the mother. A healthy 2780 g baby was delivered at 39 weeks’ gestation by vaginal delivery.

**Table 2:** Embryo screening results

Code	Break(1–3)	Break(2–6)	Break(5–9)	Break(8–7)	Break(8–1)	Break(2–5)	Break(4–7)	Break(6–9)	PGT-A result	Result
Embryo1	**√**	**√**	**√**	**√**	**√**	**√**	**√**	**√**	normal	OK
Embryo2	**×**	**√**	**√**	**√**	**√**	**√**	**√**	**×**	NA	Failed
Embryo3	**×**	**×**	**√**	**√**	**√**	**√**	**√**	**√**	NA	Failed
Embryo4	**√**	**√**	**√**	**√**	**√**	**√**	**√**	**√**	chr16 triploid	Failed
Embryo5	**×**	**×**	**√**	**√**	**√**	**√**	**√**	**√**	NA	Failed
Embryo6	**√**	**×**	**√**	**×**	**×**	**√**	**√**	**√**	NA	Failed
Embryo7	**×**	**×**	**√**	**√**	**√**	**√**	**×**	**√**	NA	Failed
Embryo8	**×**	**×**	**√**	**√**	**√**	**√**	**√**	**√**	NA	Failed
Embryo9	**×**	**×**	**×**	**×**	**×**	**×**	**×**	**×**	6q16.1 del	Failed
Embryo10	**√**	**×**	**√**	**×**	**×**	**√**	**√**	**√**	NA	Failed
Embryo11	**×**	**√**	**√**	**√**	**√**	**×**	**×**	**√**	NA	Failed

## Discussion

It has previously been demonstrated that precise characterization of apparently balanced CCRs in non-affected individuals is crucial as they are likely to produce gametes with unbalanced products because of quadrivalent formations during meiosis, which usually results in reproductive failure, recurrent miscarriages or affected offspring [20, 21].

In this study, we present a rare case of a non-affected female experienced recurrent miscarriage with CCRs. The karyotyping report indicates a balanced translation between chromosome 1 and chromosome 4 and a q25q28 fragment of chromosome 4 inserted into chromosome 5q22. However, WGL-MPS utilized in this study allowed accurate reconstruction of the derivative chromosomes, and interestingly revealed a far more complex rearrangement picture compromising translocation of three fragments of chromosome 1, a fragment of chromosome 4 and a fragment of chromosome 5. It has previously been demonstrated that cryptic deletions are a common finding in “balanced” reciprocal and complex chromosome rearrangements, which may explain the clinical phenotypes in many cases [20]. The woman in this case carried CCRs and had already experienced two miscarriages. Due to high degree of her CCRs, there was very little possibility for her to give birth to a normal child through natural pregnancy and she faced with an increased risk for having an affected offspring. After consulting with her physicians, the couple decided to go through the assisted reproduction procedure. Because of CCRs, breakpoints need to be accurately determined before transplantation, and embryos that do not have breakpoints or carry breakpoints like the mother need to be kept. The embryos retained in the above screening should be tested by PGT-A to screen out those with abnormal chromosomal structure and number. If this woman and her child reproduce in the future, they need assisted reproduction and do the above corresponding tests to screen for appropriate embryos. Our case demonstrated that WGL-MPS method combining with junction-spanning PCR and PGT-A could be a powerful and practical tool in the process of risk assessment and embryo selection for couples with recurrent miscarriage due to chromosomal abnormalities.

Precise identification of the breakpoints has been one of the most interesting and technically challenging field in cytogenetics for investigating the possible genotype and phenotypic outcomes of carriers of chromosomal rearrangements. Conventional techniques, such as in situ hybridization with fluorescent dye-labelled bacterial artificial chromosome clones and DNA array hybridization combined with chromosome sorting have been adopted to characterize the chromosome breakpoints to the kilobase level [22–25]. However, these techniques are laborious and expensive. In the recent years, massive parallel sequencing has been developed to accurately detect the breakpoints, but this technique is highly dependent on prior knowledge of the affected G-band region. In our study, we developed a practical solution which could rapidly localize the cryptic breakpoints to individual genes, and substantially improve the prediction of the fertility risks and phenotypic outcomes and timely inform antenatal medical care within a time frame that allows for clinical action. In addition, our approach which could precisely identify the breakpoints down to nucleotide level, can better assess the genotypic and phenotypic consequences of chromosomal abnormalities.

## Conclusions

Accurate breakpoints mapping is the key to provide prediction for fertility risk, genetic counseling, and fertility guidance for couples who carry CCRs. In this study, a robust approach, whole-genome low-coverage mate-pair sequencing (WGL-MPS), was applied to a female CCRs carrier without taking advantage of the result of G-banding, precisely revealed 9 breakpoints and 1 cryptic deletion related to fertility risks, and provided crucial information for the PGT-A process. Junction-spanning PCR and PGT-A were performed on the 11 embryos cultivated and only one embryo was considered qualified which carried the exactly same CCRs as the female carrier, whose phenotype was normal. The amniotic fluid was also investigated by WGL-MPS, which verified the baby carried the same CCRs. A healthy baby was delivered at 39 weeks’ gestation by vaginal delivery. Our study illustrates the WGL-MPS approach especially combining with junction-spanning PCR and PGT-A is a valuable tool in assisted reproduction for couples with complex chromosomal abnormalities and recurrent miscarriages.

## Data Availability

The datasets used and/or analyzed during the current study are available from the corresponding author on reasonable request.
